# Perceived social and built environment associations of leisure-time physical activity among adults in Sri Lanka

**DOI:** 10.1186/s13104-021-05810-6

**Published:** 2021-10-20

**Authors:** Shreenika De Silva Weliange, Madhawa Perera, Jagath Gunatilake

**Affiliations:** 1 Department of Community Medicine, University of Colombo, Colombo, Sri Lanka; 2 Department of Gynaecology and Obstetrics, Faculty of Medicine and Allied Sciences, Rajarata University of Sri Lanka, Anuradhapura, Sri Lanka; 3 Postgraduate Institute of Science, University of Peradeniya, Peradeniya, Sri Lanka; 4 Department of Geology, University of Peradeniya, Peradeniya, Sri Lanka

**Keywords:** Leisure time physical activity, Social environment, Built environment, Self-efficacy

## Abstract

**Objective:**

Although perceived neighbourhood environment is considered a predictor of leisure-time physical activity (LTPA), evidence for this is limited in South Asia. Thus, the aim was to determine the association between neighbourhood social and built environment features in carrying out LTPA among adults in Colombo District, Sri Lanka. A cross-sectional study among 1320 adults was carried out using validated questionnaires for physical activity (PA) and built environment data collection. Multiple logistic regression analysis was conducted to assess the associations between environment characteristics and LTPA after adjusting for gender, age, employment status, income level and sector of residence.

**Results:**

A total of 21.7% of adults participated in some LTPA. The commonest type of LTPA was walking; carried out by 14.5%. Moderate and vigorous activity at leisure was carried out by 10.3% and 3.9% respectively. Perceived social acceptance for PA was positively associated with LTPA. Out of the built environment characteristics perceived infrastructure for walking, and recreational facilities for PA were negatively associated with LTPA. Self-efficacy emerged as an important positive correlate of LTPA. The participants were positively influenced by the self-efficacy and perceived social environment which should be addressed when promoting LTPA.

## Introduction

Physical activity (PA) reduces the risk of non-communicable diseases (NCDs), premature mortality and increase life expectancy [1]. Although PA levels of South Asian adults are modest, participation in leisure-time physical activity (LTPA) is not sufficient [2]. This low level of LTPA was also observed in Sri Lanka [3], and requires immediate attention in order to reduce the burden of NCDs, as domestic and job-related PA has also been rapidly reducing due to urbanised lifestyles [2, 3].

Ecological approach is better suited to create active living communities as people select different places for LTPA depending on the built and social environment features [4]. Ecological model of PA describes the importance of multilevel approaches targeting individuals, social and built environments to promote PA at population level by providing safe, attractive, and convenient places, encouraging the use of those places, and changing social norms or culture [4]. Features of the built environment like walkable community designs, aesthetics, pedestrian facilities, bicycle facilities, trails and parks appear to be associated with LTPA [4, 5]. Socio-cultural attributes such as social support, social networks, socioeconomic differences, social cohesion and social capital also seem to shape one’s LTPA behaviour [6]. In addition, there is evidence that utility of certain built environment features such as parks and sports facilities for recreational purposes was also mediated by social environment [7]. Thus, influence of social and built environment characteristics in the local neighbourhoods would be a possible reason for this low level of LTPA among Sri Lankan adults.

Since people experience or perceive their neighbourhood built environment differently, objective measures of the built environment may not reflect their actual use of the environment [8, 9] for PA. From a multi-centre study, Cerin et al. [8] suggested that objective environmental conditions affect PA through one's perceptions, where safety, aesthetics and pedestrian infrastructure are key attributes. Thus, measuring and altering perceptions of people about their neighbourhood environment are more likely to affect cognitions supportive of forming intentions to be physically active [10, 11]. This is especially true for LTPA, as people are not compelled to carryout PA during their leisure unlike domestic and job-related PA.

Due to the diversity of culture, social-context, infrastructure and policies across countries [12, 13], each setting would have unique relationships between the environment and LTPA. Similarly, social factors appear to be differently affecting LTPA of Asians who live in their own countries than elsewhere [14]. Thus, additional studies beyond high-income Western and Asian populations living in Western countries are necessary to improve the generalizability of available findings and to facilitate cross-country comparisons.

Thus, the aim of this study was to assess the perceived social and built environment associations of leisure-time physical activity among adults living in Colombo District, Sri Lanka.

## Main text

### Methods

This study was a component of a community based cross sectional study conducted in the district of Colombo among a representative sample of adults aged 20–59 years living in urban and rural areas of Colombo District for a period of more than six months. The sample was selected using stratified cluster sampling probability proportionate to size of adults in a village within the Colombo district. Houses were selected randomly from each cluster followed by selection of an adult randomly. Adults with physical and mental disability or illness preventing engagement in PA, institutionalised adults, pregnant women, as well as adult visitors to the study area were excluded. A full description of the sampling procedure was published elsewhere [3].

Socio-demographic information including age, employment status, level of education, income, sector of residence and gender were assessed using an interviewer administered questionnaire.

Participants self-reported their PA using the long version of International Physical Activity Questionnaire (IPAQ-long) which is culturally adapted and validated in Sri Lanka [15]. IPAQ-long records the number of days and duration of walking, moderate and vigorous intensity PA undertaken during the previous week in different domains (job, transportation, domestic/garden chores and leisure). The PA in leisure time activity was considered for analysis and is described as median and interquartile range along with the mean and standard deviation as the data distribution of LTPA was skewed to the right. Further, the number of persons engaged in any type of LTPA for more than 10 min was described in different domains of walking, moderate activity, vigorous activity and total activity as numbers and percentages.

The physical and the social environments were assessed using Physical and Social Environment Scale (PASES). The tool was developed and validated to assess the physical and social environment factors associated with PA in Sri Lankan context [16]. This scale measures population density, land use mix diversity, infrastructure for walking, aesthetics and facilities for cycling, vehicular traffic safety, access and connectivity, recreational facilities for PA, safety, social cohesion and social acceptance of PA. Each item in the tool was described using descriptive statistics.

To assess the relationship of LTPA with neighbourhood environment and socio-demographic characteristics a crude analysis was carried out between carrying out any LTPA and the environment attributes. This was followed by a multivariate regression analysis. Gender, sector of residence, education level, monthly income, employment status and self-efficacy were added as covariates in the regression model since they are common covariates of PA in Sri Lankan adults [3]. Self-efficacy was also added as it may affect the perceptions towards the environment [10]. Odds ratios (ORs) with 95% confidence intervals were calculated and the statistical significance was set at p < 0.05. Data analysis was carried out using SPSS-17 package.

All participants responded completely to all questions and there were no missing values as the information was collected using an interviewer administered questionnaire by trained pre-intern medical officers who visited households at a time convenient to the participants, and as the questions were not very sensitive.

### Results

Most participants were in the low-income category and were currently employed; and the full description of the sample was published previously [3]. Distribution of LTPA among the participants is given in Table 1. Only 21.7% of adults participated in any LTPA and the commonest type of LTPA was walking, carried out by 14.5% of sample.

**Table 1:** Pattern of Leisure time activity of participants (N = 1320)

Activity indicator	Walking	Moderate activity	Vigorous activity	Total Leisure
Mean Minutes per week of activity (SD)	18.7 (67.1)	13.6 (57.7)	5.9 (38.2)	38.2 (114.0)
Median Minutes per week of activity (IQR)	0 (0–0)	0 (0–0)	0 (0–0)	0 (0–0)
At least some activity n (%)	191 (14.5)	136 (10.3)	52 (3.9)	287 (21.7)

The neighbourhood environment characteristics are presented in Table 2 with a higher score indicating an environment conducive for PA. Items with a median score less than 2 showed the attributes in the environment that were not conducive for PA. They are: non availability of a separate grass/sand strip for walking, pavement obstructions, non-availability of cycle lanes, not having large trees and interesting things to look at, dust and fumes in the environment, not having free or very low cost centers for sports and exercise, not having adequate street lights and a social environment factor of less encouragement for PA from the neighbours.

**Table 2:** Perceived neighbourhood environment characteristics

Neighbourhood environment characteristics (N = 1320)		Mean	Median
Infrastructure for walking	Sidewalks in the main street [Q1]	3.32 (1.426)	4 (2–4)
	***Grass/sand strip for walking in the by-roads with vehicular traffic [Q2]***	***2.23 (1.208)***	***2.0 (1–3)***
	***Pavements/ sidewalks free of obstruction [Q3]***	***2.97 (1.418)***	***2.0 (2–4***
	Pavements/ sidewalks free of hazards [Q4]	3.77 (1.208)	4.0 (3–5)
Aesthetics and facilities for cycling	***Special lanes, separated paths to cycle [Q5]***	***1.42 (0.722)***	***1.0 (1–2)***
	Shade in the pathways [Q6]	3.32 (1.229)	4.0 (2–4)
	Trees in the neighbourhood [Q7]	3.07 (1.233)	3.0 (2–4)
	***Interesting/pleasant things to look (Natural sights, beautiful buildings) [Q8]***	***2.40 (1.043)***	***2.0 (2–4)***
Vehicular traffic safety	***Free of dust and fumes [Q14]***	***2.83 (1.364)***	***2.0 (2–4)***
	Low movement of traffic [Q15]	3.06 (1.243)	4.0 (2–4)
	Low speed of vehicles [16]	3.15 (1.169)	4.0 (2–4)
	Less road traffic accidents [Q20]	3.49 (1.214)	4.0 (2–4)
Access and connectivity	Alternative routes for getting from place to place [Q9]	3.70 (0.879)	4.0 (4–4)
	Many places that need to be visited day to day are within easy walking distance [Q10]	3.65 (1.112)	4.0 (4–4)
	Short distance to main road [Q11]	3.82 (1.046)	4.0 (4–4)
	Short distance to transit stop/ public transport [Q12]	3.76 (1.050)	4.0 (4–4)
	Terrain is not hilly and good for walking [Q13]	4.12 (1.033)	4.0 (4–4)
	Presence of pedestrian crossing, signals and overhead bridges [Q24]	3.09 (1.244)	4.0 (2–4)
Recreation facilities for PA	Free or very low cost centres for sports and exercise [Q17]	2.88 (1.137)	3.0 (2–4)
	Public spaces for sport and recreation (Park, beach) [Q18]	3.15 (1.182)	4.0 (2–4)
	Easy access to the public places for sports and recreation [Q19]	3.11 (1.188)	4.0 (2–4)
Safety	Low crime rate[Q21]	3.35 (1.205)	4.0 (2–4)
	***Well-lit roads [Q22]***	***2.61 (1.173)***	***2.0 (2–4)***
	Free of stray animals [Q23]	3.26 (1.179)	4.0 (2–4)
Social cohesion	Harmony between people in the neighbourhood [Q25]	3.68 (0.948)	4.0 (3–4)
	Respect among persons in the neighbourhood [Q26]	3.46 (0.965)	4.0 (3–4)
	Free of social disorder/ social disputes [Q27]	3.57 (0.889)	4.0 (3–4)
	Helpful people in the neighbourhood [Q28]	3.83 (0.745)	4.0 (4–4)
	Trustworthy people in the neighbourhood [Q29]	3.43 (0.927)	4.0 (3–4)
	Good interactions between people in the neighbourhood [Q30]	3.28 (1.016)	4.0 (2–4)
Social acceptance of PA	People in the neighbourhood physically active [Q31]	2.86 (1.134)	3.0 (2–4)
	***The people encourage each other for active living [Q32]***	***2.32 (0.940)***	***2.0 (2–3)***
	Social acceptance for being active for day to day activities [Q33]	3.20 (0.997)	3.0 (2–4)
	social acceptance for walking, exercising and recreational PA [Q34]	2.87 (1.004)	3.0 (2–4)
Composite land use mix and accessibility score		3.33 (0.813)	3.36 (2.8–4.0)

Table 3 shows that after adjusting for covariates, being male (OR = 4.36; CI = 2.99–6.36), living in urban areas (OR = 1.81; CI = 1.11–2.94), higher education (OR = 1.59; CI = 1.12–2.25), higher income (OR = 1.61; CI = 1.13–2.29) and those with higher perceived self-efficacy for PA (OR = 1.85; CI = 1.50–2.28) were significantly associated with carrying out some LTPA. Further, social acceptance for PA was associated with higher LTPA (OR = 2.14; CI = 1.65–2.77) while age was inversely associated with LTPA. Although, perception of the infrastructure for walking (OR = 0.44; CI = 0.25–0.77), and recreational facilities for PA (OR = 0.80; CI = 0.68–0.96) showed significant relationship to LTPA, the direction of the association was reversed to what was expected.

**Table 3:** Leisure time physical activity and its association with socio-demographic and environment characteristics

	Unadjusted Odds ratio (95% CI)	Adjusted Odds ratio (95% CI)	
Environment and socio-demographic characteristics			
Infrastructure for walking	0.42^*^ (0.26–0.68)	0.44^*^ (0.25–0.77)	
Aesthetics and facilities for walking	1.26* (1.04–1.53)	0.87 (0.66–1.13)	
Vehicular traffic safety	0.90 (0.79–1.02)	1.14 (0.90–1.44)	
Access and connectivity	0.89 (0.73–1.07)	0.91 (0.67–1.22)	
Recreation facilities for PA	0.79* (0.69–0.90)	0.80* (0.68–0.96)	
Safety	0.79* (0.68–0.92)	0.87 (0.70–1.09)	
Social cohesion	1.36* (1.07–1.74)	0.99 (0.73–1.33)	
Social acceptance of PA	1.97* (1.62–2.40)	2.14* (1.65–2.77)	
Land use mix score	0.96 (0.82–1.13)	1.23 (0.95–1.59)	
*Residential density*			
Annexed/ row houses/ 2–3 story flats	0.68 (0.49–0.95)	0.67 (0.40–1.12)	
Mix of detached and annex houses	1.72* (1.15–2.59)	1.41* (0.81–2.46)	
Apartment 4-stories or above	0.61 (0.23–1.60)	1.29 (0.37–4.45)	
Detached single family	Reference	Reference	
Age in years	0.98* (0.97–0.99)	0.97* (0.96–0.98)	
*Gender*			
Male	3.70* (2.78–4.92)	4.36* (2.99–6.36)	
Female	Reference	Reference	
*Sector of residence*			
Urban	2.00* (1.43–2.81)	1.81* (1.11–2.94)	
Rural	1.74* (1.20–2.52)	1.32 (0.74–2.37)	
CMC	Reference	Reference	
*Education Level*			
Passed G.C.E A/L or higher	2.48* (1.89–3.25)	1.59* (1.12–2.25)	
Education less than G.C.E. A/L	Reference	Reference	
*Average monthly income in Rupees (USD)*			
> 30,000 (230)	2.39* (1.81–3.16)	1.61* (1.13–2.29)	
< = 30,000 (> 230)	Reference	Reference	
*Work status*			
Employed	2.41 (1.81–3.20)	0.78 (0.53–1.15)	
Not employed	Reference	Reference	
Self-efficacy score	2.01* (1.70–2.42)	1.85* (1.50–2.28)	
Constant		0.14	

Obtained from regression models with mutual adjustments of the factors listed in the Table

* Indicate those that are statistically significant at p < 0.05

### Discussion

This study assessed the perceived social and built environment associations of LTPA among adults living in Colombo District, Sri Lanka. The results revealed that males, urban dwellers, young adults, people with higher education and income have been carrying out some LTPA compared to their counterparts. Although perceptions of infrastructure for walking and recreational facilities were not positively associated with accumulating at least some LTPA, social acceptance and self-efficacy were seen to be positively associated.

Surprisingly, it was found that most of the built environment features were not significantly associated with LTPA, and in particular, perceptions of infrastructure for walking and recreational facilities were inversely related to LTPA. These inverse relationships might be due the fact that people who do at least some LTPA are more aware of the quality, accessibility, condition and usability of available infrastructure and facilities in their neighbourhood, rather than people who are not involve in any LTPA. Further, poor satisfaction of available infrastructure and facilities might limit participation in LTPA [4] and drive people to use facilities far from their neighbourhoods. Supporting this, studies have also shown that apart from poor conditions, amenities [17], location [18] and perceived incivilities in the community also directly hinder utility of public recreational facilities [19]. Thus, importance of maintaining quality of recreational facilities and designing facilities complying with specific needs and recreational preferences of a community are emphasized. Sri Lankans are more active in domestic and job-related PA [3] for which built environment is less relevant, and this may be another reason for the observed poor relationship between built environment and LTPA. Our findings are in contrast to evidence from Western countries [20] and other Asian countries [21, 22]. Our findings suggest that built environment features in typical activity-friendly environments alone do not motivate LTPA of Sri Lankan adults, possibly owing to the unique socio-cultural elements that intervene in making a person active at leisure.

In contrast to Western cultures where LTPA has been increasing [23], social acceptance for LTPA is normally low in South Asian countries where people count regular household chores, caring of children and manual labour as physical exercise [24–26]. As a result, LTPA has not been seen as a requirement in South Asian communities and a majority do not feel motivated to be active during their leisure [27]. This belief may be related to socio-cultural background in South Asia [2], or may relate to lack of knowledge about the benefits of LTPA [19, 26]. In some South Asian cultures, spending time for LTPA was considered as selfish [28, 29], while doing outdoor activities, regular exercise and attending gyms were considered weird [24–26]. Thus, in most South Asian cultures, including the current study sample, the society encourages people to be active within day-to-day activities [25, 26, 28]. However, it was seen in the present study that the odds of having some LTPA was significantly higher with those who perceived having higher social acceptance for LTPA in their societies. Further social acceptance was observed as the most influential correlate of LTPA in our sample. Strong family, societal, and religious ties observed among the Sri Lankans may be the reason for the requirement of social acceptance to involve in LTPA, which otherwise may be considered as culturally inappropriate behaviour. Thus, LTPA promotion efforts in Sri Lanka should focus on gaining social acceptance for LTPA, which would mitigate social barriers such as feeling embarrassed of being seen by others while exercising in public places [26, 27], wearing specific garments while performing exercise [28] and judged negatively by others at recreational places [19].

Self-efficacy has been identified as a strong predictor of leisure time exercise behaviour in adults [30]. Self-efficacy is a form of cognitively based motivation that is described as the confidence in one's own ability to successfully execute a particular behavior [31]. The choice of behavior, amount of effort, and sustainability are depend on the person’s self-efficacy that relies on performance accomplishment, vicarious experience, verbal persuasion and emotional arousal [31]. Self-efficacy also facilitates initiating and continuing exercise [32]. For example, self-efficacy was a predictor of initiating PA among women with gestational diabetes mellitus who received obstetric care at a teaching hospital in Sri Lanka [33]. Studies have also shown that self-efficacy mitigates the constraints of living in poor walkable environments, towards being physically active at leisure [34, 35]. Although self-efficacy has been widely acknowledged as a critical factor of promoting LTPA in Western countries [30, 35, 36], this is one of the few to demonstrate that self-efficacy is a positive correlate of LTPA among Sri Lankan adults. The findings of the current study comply with Social Learning Theory [31]. Collectively, the results of this study suggest that interventions improving LTPA should primarily focus on improving self-efficacy. Infrastructure and recreational facilities might be less important to people if the people are motivated to carry out LTPA.

Social acceptance and self-efficacy can be considered the most influential factors for facilitating LTPA among Sri Lankan adults. Although the perception of built environment features were not positively associated, it might be interesting to look also at the objective measurements together at the same time to understand these complex relationships. However, the findings open up the discussion of built environment influences to PA in a South Asian setting.

## Limitations

Self-reported questionnaire was used to quantify PA, which may be affected by recall and social desirability bias. The sample was drawn from only one district in Sri Lanka which further precludes the generalizability of the results. Additionally, the cross-sectional nature of our study cannot prove a causal association between neighbourhood environment and LTPA.

## Data Availability

The datasets used and/or analysed during the current study are available from the corresponding author on reasonable request.

## Abbreviations

IPAQ: International Physical Activity Questionnaire
LTPA: Leisure-time physical activity
NCDs: Non-communicable diseases
PA: Physical activity
